# Poloxamer 407 Induces Hypertriglyceridemia but Decreases Atherosclerosis in Ldlr^−/−^ Mice

**DOI:** 10.3390/cells11111795

**Published:** 2022-05-30

**Authors:** Xueying Peng, Zeqin Lian, Xiao-Yuan Dai Perrard, Yunjie Xiao, Jing Ni, Veronica O’Brien, Henry Dong, Henry J. Pownall, Christie M. Ballantyne, Huaizhu Wu

**Affiliations:** 1 Key Laboratory of Clinical Cancer Pharmacology and Toxicology Research of Zhejiang Province, Department of Clinical Pharmacology, Affiliated Hangzhou First People’s Hospital, Zhejiang University School of Medicine, Hangzhou 310006, China; xueyingpeng@zju.edu.cn; 2 Department of Medicine, Baylor College of Medicine, One Baylor Plaza, MS BCM285, Houston, TX 77030, USA; zeqin.lian@gmail.com (Z.L.); xdai@bcm.edu (X.-Y.D.P.); jessiexiao6780@gmail.com (Y.X.); 15900750278@163.com (J.N.); brien@bcm.edu (V.O.); cmb@bcm.edu (C.M.B.); 3 College of Natural Science, University of Texas at Austin, Austin, TX 78712, USA; 4 Department of Cardiology, Shanghai Tenth People’s Hospital, Tongji University School of Medicine, Shanghai 200072, China; 5 Department of Biochemistry, University of Houston, Houston, TX 77204, USA; 6 Department of Pediatrics, Children’s Hospital of Pittsburgh UPMC, University of Pittsburgh School of Medicine, Pittsburgh, PA 15224, USA; dongh@pitt.edu; 7 Center for Bioenergetics, Houston Methodist Research Institute, Houston, TX 77030, USA; hjpownall@houstonmethodist.org

**Keywords:** poloxamer 407, atherogenesis, hypertriglyceridemia, foamy monocytes, lipid metabolism, monocyte phenotype

## Abstract

Background: Hypertriglyceridemia (HTG) increases the risk for atherosclerotic cardiovascular disease, but underlying mechanisms are incompletely understood. Circulating monocytes play an important role in atherogenesis by infiltrating arterial walls, where they differentiate into macrophages. We tested the hypothesis that HTG is mechanistically linked to atherogenesis by altering the monocyte phenotype and infiltration into atherosclerotic lesions in a model of diet-induced atherogenesis in Ldlr^−/−^ mice. Methods: HTG was induced in male Ldlr^−/−^ mice, fed a Western, high-fat high-cholesterol diet, by daily injection of poloxamer 407 (P407), a lipoprotein lipase inhibitor, for seven weeks. Atherosclerosis, monocyte phenotypes, and monocyte migration into atherosclerotic lesions were determined by well-validated methods. Results: Compared with the saline control, P407 injection in Ldlr^−/−^ mice rapidly induced profound and persistent HTG, modestly elevated plasma cholesterol levels, and increased levels of triglyceride and cholesterol carried in very-low-density lipoprotein and low-density lipoprotein. Unexpectedly, mice receiving P407 versus saline control showed less atherosclerosis. Following induction of HTG by P407, CD36^+^ (also CD11c^+^), but not CD36^−^ (CD11c^−^), monocytes showed early increases in lipid accumulation, but the number of CD36^+^ (not CD36^−^) monocytes was dramatically decreased afterwards in the circulation until the end of the test. Concurrently, CD36^+^ (CD11c^+^) monocyte migration into atherosclerotic lesions was also reduced in mice receiving P407 versus controls. Conclusions: P407 induced severe HTG, but reduced atherosclerosis, in Ldlr^−/−^ mice, possibly because of profound reductions of circulating CD36^+^ (CD11c^+^) monocytes, leading to decreased monocyte migration into atherosclerotic lesions.

## 1. Introduction

Atherosclerotic cardiovascular disease (ASCVD) is the leading cause of death in the US and globally. Besides hypercholesterolemia, hypertriglyceridemia (HTG), with elevated triglyceride (TG)-rich lipoproteins (mainly very-low-density lipoprotein (VLDL), chylomicrons (CM), and their remnants), has emerged as an important causal ASCVD risk factor [1]. A large number of epidemiologic studies have shown an association of HTG and ASCVD risk [2]. Genetic studies also showed a casual role of HTG in ASCVD [3,4,5,6]. Statin-treated individuals with HTG still have a higher ASCVD risk than those with normal TG levels [7]. However, the mechanism(s) by which HTG increases ASCVD risk remain incompletely understood.

Circulating monocytes play important roles in atherogenesis by infiltrating the arterial walls, where they differentiate into macrophages that accrete lipids, thereby forming foam cells. Hypercholesterolemia, which is associated with increased numbers of circulating monocytes, alters monocyte phenotypes, leading to increased monocyte infiltration into arterial walls, which supports and accelerates atherogenesis [8,9,10,11,12]. We have reported that hypercholesterolemia in mice induces formation of circulating foamy monocytes, which contain intracellular lipid droplets [8,12]. Of note, lipid accumulation in monocytes is not monodispersed, and foamy monocytes (those with abundant lipid droplets) account for ~50% of the total monocytes in hypercholesterolemic mice [8,12]. In the same mice, “non-foamy” monocytes, which are lipid-poor, are mostly CD36^−^CD11c^–^, whereas foamy monocytes express high levels of CD36, a scavenger receptor, and CD11c, a β2 integrin that mediates monocyte adhesion and may confer a high potential for foamy monocytes to invade the arterial walls and accelerate atherogenesis [8,12]. Indeed, foamy monocytes infiltrate into plaques and play pivotal roles in atherogenesis in mice with hypercholesterolemia [8,12]. Importantly, foamy monocytes occur in humans with familial hypercholesterolemia and may be mechanistically linked to premature ASCVD in these patients [13,14]. Notably, HTG also increases lipid accumulation in monocytes in humans [15,16,17,18,19,20]. However, whether foamy monocytes contribute to atherosclerosis in HTG remains to be determined.

In the present study, we used Ldlr^−/−^ mice with HTG induced by poloxamer 407 (P407), an inhibitor of lipoprotein lipase (LPL), [21,22] to investigate the effects of HTG on atherosclerosis and the potential role of monocytes. As expected, P407 injection into Ldlr^−/−^ mice rapidly and profoundly increased plasma TG levels, modestly elevated cholesterol levels, and increased the TG and cholesterol associated with VLDL and LDL, effects that persisted throughout P407 treatment. Unexpectedly, compared with the saline control, P407 injection for seven weeks reduced atherosclerosis in Ldlr^−/−^ mice. P407 injection rapidly increased lipid accumulation in circulating monocytes, particularly CD36^+^ monocytes, and elevated CD11c levels in CD36^+^ monocytes. Strikingly, in P407-treated Ldlr^−/−^ mice, profound reductions in the number of circulating CD36^+^ (also CD11c^+^) monocytes persisted throughout P407 treatment, during which there was also less CD36^+^ monocyte infiltration into atherosclerotic lesions. Thus, less monocyte infiltration into atherosclerotic lesions may be mechanistically linked to the reduction in atherosclerosis in P407-treated versus saline control mice.

## 2. Materials and Methods

The data that support the findings of this study are available from the corresponding author upon reasonable request. Please see the Major Resources Table S1 in the Supplementary Materials.

### 2.1. Animals, Diets, and P407 Injection

All mouse experiments were approved by the Institutional Animal Care and Use Committee of Baylor College of Medicine. Ldlr^−/−^ mice were initially purchased from The Jackson Laboratory (stock number: 002207) and maintained in a light- and temperature-controlled pathogen-free animal facility at Baylor College of Medicine. To avoid the potential confounding effects of estrogen on lipid metabolism and atherosclerosis development, [23,24,25] male mice were used. After weaning, mice were fed a normal laboratory diet (Rodent Diet 5010; LabDiet, St. Louis, MO, USA) and, at age 8 weeks, switched to a Western, high-fat high-cholesterol diet (WD, which contained 21% milkfat (*w*/*w*) and 0.2% cholesterol; Dyet 112734, Dyets, Inc., Bethlehem, PA, USA) and maintained on the WD until the end of experiments. Concurrent with WD feeding, mice received intraperitoneal injection of P407 (0.25 g/kg in 0.2 mL of sterile saline, Pluronic F-127, Sigma-Aldrich, Inc., St. Louis, MO, USA) or the same volume of sterile saline alone every other day [21,22]. Blood was collected from the facial vein at time points indicated in the Results Section for FACS analysis of monocytes and assays of total TG and cholesterol levels by enzymatic methods using commercial kits (Wako Diagnostics). Mice were sacrificed at 7 weeks of P407 or saline alone injection. Blood was collected by cardiac puncture under deep anesthesia for plasma isolation and lipoprotein profiling, after which aortas and hearts (also livers and epididymal adipose tissue) were collected for the analysis of atherosclerosis.

In separate experiments, male Ldlr^−/−^ mice were fed the WD and received injections of P407 or saline alone at different time points for monitoring monocyte infiltration into atherosclerotic lesions (see below).

In addition, 8-week-old male Ldlr^−/−^ mice with human apoCIII transgenic expression [26] (apoCIIItgLdlr^–/–^) were fed the WD for 7 weeks, and monocytes were examined by FACS analysis.

### 2.2. Plasma Lipoprotein Profiling

Plasma lipoproteins were fractionated by size-exclusion chromatography (SEC, Superose HR6 column, GE Health) as previously described [27]. Cholesterol and TG concentrations of each fraction were determined enzymatically using commercial kits (Wako Diagnostics) and expressed as mg/dL in each fraction or percent of TG and cholesterol of each fraction in total fractions [27].

### 2.3. Antibodies and FACS Analysis of Circulating Monocytes

FACS analysis of circulating monocytes was performed as previously described [8,12,27]. Briefly, 50 μL of blood was diluted with an equal volume of PBS and incubated with fluorescence-conjugated monoclonal antibodies against CD115 (PE-conjugated, eBioscience; or PE/Cy7-conjugated, Biolegend), CD36 (FITC-conjugated, Bio-Rad Laboratories), or CD11c (PerCP-Cy5.5-conjugated, eBioscience; or APC-conjugated, Biolegend) for 20 min in the dark. Samples were washed once with PBS and incubated with BD FACS Lysing Solution (BD Biosciences) for 20 min to lyse red blood cells. After being washed two more times with PBS, samples were resuspended in 2% paraformaldehyde in PBS for FACS analysis. For Nile red staining, after red blood cells were lysed, samples were stained with Nile red (0.1 μmol/L, Invitrogen) for 20 min, and then the samples were washed twice with PBS and finally resuspended in 2% paraformaldehyde in PBS for analysis. Monocyte apoptosis was examined in diluted blood samples using the Annexin V-FITC/propidium iodide (PI) Apoptosis Detection kit (BD Biosciences) along with PE-Cy7-conjugated anti-CD115 and APC-conjugated anti-CD11c monoclonal antibodies. Data were collected by a BD LSRII cytometer and analyzed using Kaluza (Beckman Coulter) or FlowJo software (Tree Star Inc.). CD115^+^ leukocytes were defined as total monocytes, and monocyte subsets were identified based on CD115, CD11c, and/or CD36 expression [12,27].

### 2.4. Cell Culture

THP1 cells (ATCC, Manassas, VA) were cultured in RPMI-1640 medium supplemented with 30% (*v*/*v*) plasma from Ldlr^−/−^ mice injected with P407 or saline or mixed plasma from Ldlr^−/−^ mice injected with P407 and wild-type (WT) mice (to match the TG level in the saline group). After 48 h, THP1 cells were collected and examined by FACS analysis for lipid accumulation.

### 2.5. Analysis of Monocyte Trafficking into Atherosclerotic Lesions

Two protocols were used. In protocol 1, 8-week-old Ldlr^−/−^ mice were fed the WD for ~8 weeks to induce atherosclerosis and then started receiving intraperitoneal injection of P407 in saline or saline alone every other day. Simultaneous with the first P407 or saline injection, a bolus of fluorescent microbeads (Fluoresbrite^®^ YG Carboxylate Microspheres, Polysciences, Inc., Brentwood, TN, USA) was injected intravenously into mice to specifically label CD36^+^ (CD11c^+^ and Ly-6c^low^) monocytes [12,28]. In protocol 2, 8-week-old Ldlr^−/−^ mice were fed the WD while concurrently receiving intraperitoneal injections of P407 in saline or saline alone every other day for ~8 weeks, after which they were injected with a bolus of fluorescent microbeads to label CD36^+^ monocytes. About 5 days after fluorescent microbead injection in both protocols, mice were sacrificed and CD36^+^ monocyte infiltration into atherosclerotic lesions was examined [12,28].

### 2.6. Analysis of Atherosclerotic Lesions

For the mouse atherosclerosis study, we adhered to the guidelines for experimental atherosclerosis studies as described in the American Heart Association Statement [29]. Briefly, aortas were collected and fixed in 4% paraformaldehyde and then pinned longitudinally on a strong gel pad, followed by removal of outside connecting tissue and fat. After being cut longitudinally, whole aortas were stained with oil red O (0.5 g/100 mL 60% 2-propanol, SigmaAldrich) for 10 min and washed 3 times with 60% isopropanol. Digital images were captured with a Nikon camera. Atherosclerotic plaque areas that were oil red O-positive were quantitated using ImageJ analysis software and expressed as percentage relative to the whole aorta or the brachiocephalic artery (BCA) [27].

To analyze atherosclerosis in the aortic root, mouse hearts were harvested, embedded in optimal cutting temperature medium (Sakura Finetek), and sectioned using a freezing microtome (Leica CM3050 S). Sections were collected at 5 µm/section when all 3 aortic valves appeared and stained with oil red O as described above. To examine macrophages, the sections were stained with FITC-conjugated anti-Mac-3 (BD Biosciences) and PE-conjugated anti-CD11c (eBioscience) monoclonal antibodies as described previously [27]. Digital images were captured by a Nikon camera and analyzed using Image J software [8,12,27,30]. Imaging parameters and software set-up were constant for all photomicrograph acquisitions in an experiment. Six sections with 200 µm intervals from the aorta root (whole length of aortic valve) were used for quantification of the atherosclerotic plaque size. The lumen side was selected as the region of interest, which was recognized as the space inside elastic fibers of the aorta. The area, of which the color intensity was higher than the threshold justified by the negative control section, was selected as the positive staining region [27,29]

### 2.7. Analysis of CD11c mRNA in the Liver

Total RNA was isolated from liver tissues using TRIzol Reagent (Thermo Fisher Scientific). CD11c mRNA was examined by quantitative reverse transcription polymerase chain reaction using predesigned primers and probes (Thermo Fisher Scientific) and expressed as relative levels to the 18S rRNA internal control.

### 2.8. Statistical Analysis

Statistical analyses were performed in GraphPad Prism 8.3 or higher (GraphPad Software). Scatter dot-plots and error bars were presented as mean ± SEM or median with 95% confidence interval of biological replicates. The Mann–Whitney test was used for comparison between 2 groups with *n* ≤ 5 samples in either group or for data that were not normally distributed or did not have equivalence of variance. Otherwise, the unpaired Student’s *t*-test or one-way ANOVA followed by multiple pairwise comparisons test was used for 2-group or ≥3-group comparisons. For comparisons between treatment and control groups over time or across lipoprotein fractions, two-way ANOVA with repeated measures and assumption of inequivalence of variance followed by Šídák’s multiple pairwise comparisons test was used. The differences were considered statistically significant at *p* ≤ 0.05.

## 3. Results

### 3.1. P407 Induced Rapid and Persistent Severe HTG in Ldlr^−/−^ Mice Fed WD

Administration of P407 compared to the vehicle control causes severe HTG without changing body weights in WT mice on a normal laboratory diet [21,22]. We observed that WD feeding induced weight gain in both P407- and saline-injected Ldlr^−/−^ mice. Compared to the saline control, P407 injection into Ldlr^−/−^ mice fed the WD slightly reduced weight gain (Supplementary Figure S1). At seven weeks of P407 injection, Ldlr^−/−^ mice tended to have a lower body weight and had smaller epididymal fat pads compared to the saline controls (Supplementary Figure S1).

**Figure 1:**
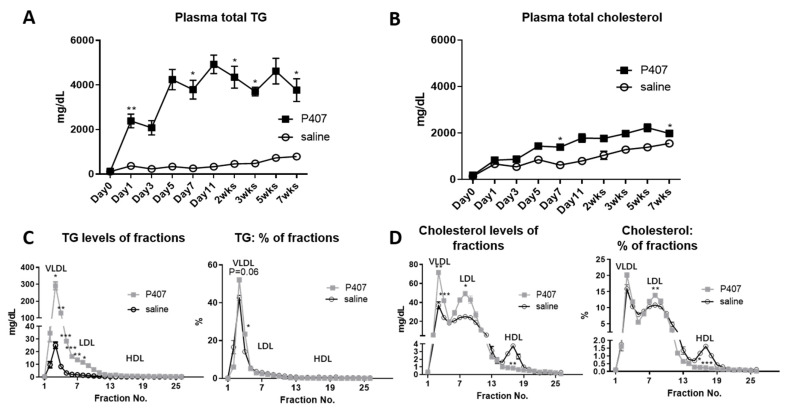
Plasma lipid levels of Ldlr^−/−^ mice on the Western diet with injection of P407 or saline control. Ldlr^−/−^ mice were fed the Western diet and injected with P407 or saline every other day. Plasma levels of total (**A**) TG and (**B**) cholesterol were measured in mice just prior to (day 0) and during P407 or saline injection. At 7 weeks of P407 or saline injection, (**C**) TG and (**D**) cholesterol distributions of lipoprotein fractions were analyzed by SEC analysis. *n* = 7 mice/group. Values are mean ± SEM. Two-way ANOVA with repeated measures and assumption of inequivalence of variance followed by Šídák’s multiple pairwise comparisons test was used for statistical analysis. * *p* < 0.05, ** *p* < 0.01, and *** *p* < 0.001 compared to saline controls.

Analysis of plasma lipid levels showed that in Ldlr^−/−^ mice fed the WD, TG levels were sharply increased from the pre-injection value of 121.6 to 2317.6 mg/dL at 24 h after the first P407 injection and remained at ~4000 mg/dL from day 5 until the end of the 7-week P407 injection schedule. Starting 24 h after the first injection, TG levels were >4.8-fold higher in Ldlr^−/−^ mice with P407 than with the saline control (Figure 1A). From day 5, plasma cholesterol levels in P407-treated Ldlr^−/−^ mice were also modestly higher than those of controls (Figure 1B).

Analysis of the SEC fractions revealed that TG levels in VLDL and LDL were higher in Ldlr^−/−^ mice receiving P407 versus the saline control (Figure 1C). As expected, in both control and P407 groups, most TG was in VLDL (and CM), whereas analysis of TG distributions across all lipoproteins showed higher TG levels in VLDL in the P407 group than in the control group (Figure 1C). Compared with controls, Ldlr^−/−^ mice receiving P407 also had higher cholesterol levels in both VLDL and LDL. Analysis across the SEC profiles showed greater cholesterol enrichment of LDL than high-density lipoprotein in the P407 group compared to the controls (Figure 1D).

Therefore, injection of P407 compared with the saline control in Ldlr^−/−^ mice fed the WD induced severe HTG and modest hypercholesterolemia, with increased TG and cholesterol levels occurring in both VLDL and LDL.

### 3.2. Ldlr^−/−^ Mice with P407 Injection Had Reduced Atherosclerosis

**Figure 2:**
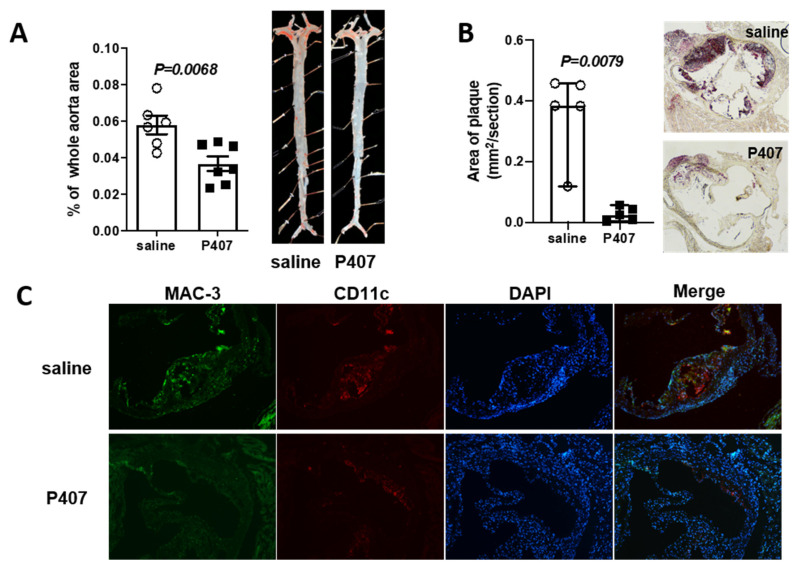
Ldlr^−/−^ mice with P407 injection had reduced atherosclerosis. Ldlr^−/−^ mice were fed the WD and simultaneously injected with P407 or the saline control and were sacrificed at 7 weeks of WD and P407 or saline injection. Atherosclerotic plaque size was quantified in (**A**) whole aortas and (**B**) aortic sinus areas after oil red O staining. Data are presented as (**A**) mean ± SEM or (**B**) median with 95% confidence interval. Unpaired Student’s *t*-test (**A**) or Mann–Whitney test (**B**) was used for statistical analysis. (**C**) Representative images of macrophages in aortic sinus atherosclerotic plaques after staining for Mac-3 (green), CD11c (red), and DAPI (blue).

Based on the role of HTG and elevated cholesterol in ASCVD, we then examined atherosclerosis in Ldlr^−/−^ mice with P407 injection. To our surprise, despite severe HTG and elevated plasma cholesterol levels, Ldlr^−/−^ mice receiving P407 for seven weeks compared with controls had significantly less atherosclerosis, as evidenced by the reduced plaque size in whole aortas, aortic sinus (Figure 2A,B), and BCA (Supplementary Figure S2) examined by oil red O staining. Mac-3^+^/CD11c^+^ foam macrophages were also lower in atherosclerotic plaques of Ldlr^−/−^ mice receiving P407 versus the saline control (Figure 2C). Thus, the reduction in foam macrophages may be mechanistically linked to the smaller atherosclerotic lesion size in the P407-treated mice.

### 3.3. P407-Induced HTG Rapidly Increased Lipid Accumulation and CD11c Levels in CD36^+^ Monocytes in Ldlr^−/−^ Mice

Based on our previous studies showing the emergence of foamy monocytes in mice with hypercholesterolemia and their contributions to atherogenesis, [8,12] we assessed lipid accumulation, indicated by the FACS side scatter (SSC, representing cell granularity and accumulation of lipid droplets) with Nile red staining, and the phenotypes of circulating monocytes in Ldlr^−/−^ mice receiving P407 and the saline control.

**Figure 3:**
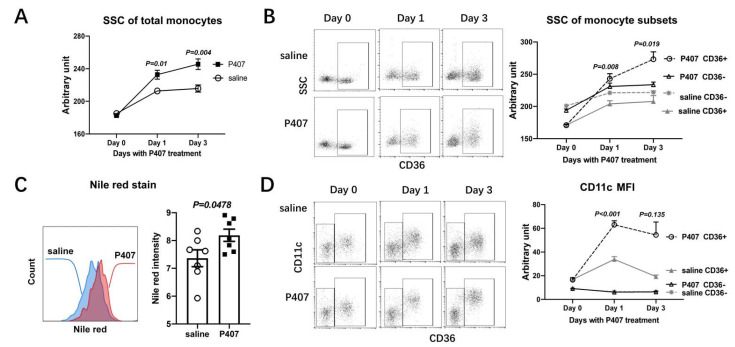
Increased lipid accumulation and CD11c levels in CD36^+^ monocytes of Ldlr^−/−^ mice with P407 injection. Changes in side scatter (SSC), indicating lipid accumulation, of (**A**) circulating monocytes and (**B**) monocyte subsets defined by CD36 in Ldlr^−/−^ mice prior to (day 0) and on days 1 and 3 of P407 or saline injection. (**C**) Nile red staining for lipids of circulating CD11c^+^ (also CD36^+^) monocytes in Ldlr^−/−^ mice on day 1 of P407 or saline injection. (**D**) Changes in CD11c levels indicated by mean fluorescent intensity (MFI) of monocyte subsets in Ldlr^−/−^ mice prior to (day 0) and on days 1 and 3 of P407 or saline injection. *n* = 5–7 mice/group. Data are shown as mean ± SEM and were analyzed by two-way ANOVA with repeated measures and assumption of inequivalence of variance, followed by Šídák’s multiple pairwise comparisons test (**A**,**B**,**D**) or by the unpaired Student’s *t*-test (**C**). *p*-values indicate comparisons of (**A**) monocytes or (**B**–**D**) the same monocyte subsets between the P407 group and controls.

At 24 h after the first injection, with the dramatic elevations in plasma TG levels (Figure 1A), Ldlr^−/−^ mice receiving P407 versus the saline control had significantly higher SSC values of monocytes (Figure 3A and Supplementary Figure S3), indicating that HTG induced by P407 induced further lipid accumulation in monocytes in Ldlr^−/−^ mice, which were hypercholesterolemic. On day 3, monocyte SSC values were consistently higher, indicating more lipid accumulation in monocytes, in Ldlr^−/−^ mice with P407-induced HTG than in the controls (Figure 3A). Further analysis revealed that on days 1 and 3, compared with controls, in P407-induced HTG, CD36^+^ monocytes (which were also mostly CD11c^+^), but not CD36^−^ (CD11c^–^) monocytes, had elevated SSC (Figure 3B), indicating more lipid accumulation confirmed by Nile red staining (Figure 3C), suggesting that HTG induced by P407 in Ldlr^−/−^ mice enhanced lipid accumulation in CD36^+^, but not CD36^–^, monocytes [8,12,27]. Importantly, along with the increased lipid accumulation, CD36^+^ monocytes in Ldlr^−/−^ mice with P407-induced HTG (for 1 and 3 days), compared with controls, expressed even higher levels of CD11c, as indicated by mean fluorescent intensity (Figure 3D). Therefore, P407 injection in Ldlr^−/−^ mice induced rapid severe HTG and concomitant early increases in lipid accumulation and CD11c levels in CD36^+^, but not CD36^–^, monocytes.

These data also indicated that despite LPL inhibition, P407 may not repress HTG-induced monocyte lipid accumulation. To confirm this, we examined effects on THP1 cell lipid accumulation of plasma from Ldlr^−/−^ mice injected with P407 or saline. As shown in Supplementary Figure S4, plasma from P407-injected Ldlr^−/−^ mice compared to that from saline control mice induced significantly more lipid accumulation in THP1 cells after culture for 48 h. At the same TG levels, plasma from P407-injected Ldlr^−/−^ mice compared to plasma from saline control mice induced similar levels of lipid accumulation in THP1 cells, consistently supporting that HTG increased monocyte lipid accumulation and that P407 per se did not suppress monocyte lipid uptake.

### 3.4. P407 Severely Reduced the Proportion of Circulating CD36^+^ Monocytes Early and Persistently in Ldlr^−/−^ Mice

**Figure 4:**
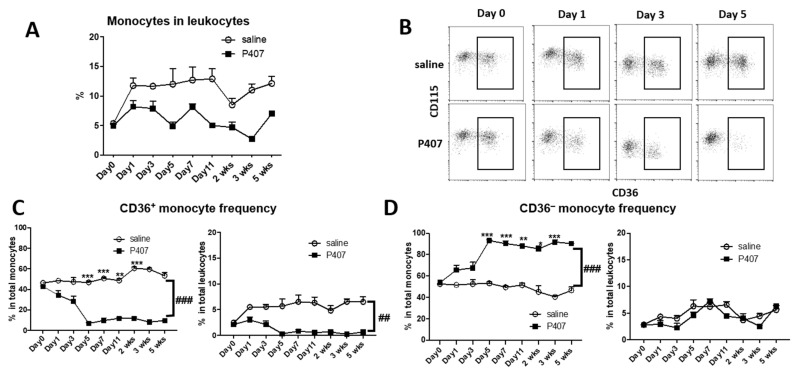
Changes in the proportions of monocytes and subsets in Ldlr^−/−^ mice with P407 injection. (**A**) Changes in percentages of total monocytes in total leukocytes in Ldlr^−/−^ mice with injection of P407 or saline control. (**B**) FACS examples showing rapid and profound reductions in CD36^+^, but not CD36^–^, monocytes in Ldlr^−/−^ mice with P407 injection. Changes in percentages of (**C**) CD36^+^ and (**D**) CD36^−^ monocytes in total monocytes or in total leukocytes in Ldlr^−/−^ mice with injection of P407 or the saline control. *n* = 7 mice/group. Data are shown as mean ± SEM and were analyzed by two-way ANOVA with repeated measures and assumption of inequivalence of variance, followed by Šídák’s multiple pairwise comparisons test. * *p* < 0.05, ** *p* < 0.01, and *** *p* < 0.001 for comparisons between P407 and control groups. ^##^ *p* = 0.01 and ^###^ *p* < 0.001 for group x time interaction by two-way ANOVA.

A previous report showed that injection of P407 in WT mice profoundly reduced Ly-6c^low^ monocytes, [21] which corresponded mostly with CD36^+^ monocytes [8,12,27]. We therefore examined the proportions of monocytes and subsets in Ldlr^−/−^ mice with P407 injection and observed that injection of P407, compared with the saline control, in Ldlr^−/−^ mice reduced the percentages of total monocytes in total leukocytes (Figure 4A). Further analysis showed that CD36^+^ monocytes were dramatically reduced in the circulation of Ldlr^−/−^ mice with P407 injection, an effect that began early and persisted throughout the injection period (Figure 4B,C), whereas the percentages of CD36^−^ (corresponding to Ly-6c^high^) [12] monocytes in total leukocytes were largely unchanged (Figure 4B,D). In Ldlr^−/−^ mice, the percentages of CD36^+^ monocytes in total leukocytes fell from ~2.1% before P407 injection to ~0.3% on day 5 after P407 injection and remained at ~0.2–0.8% (versus ~5–6.5% with saline injection) until the end of the experiment (Figure 4C). Starting from day 5, the percentages of CD36^+^ monocytes in total monocytes were <15% with P407 injection (versus ~50–60% with saline injection), and >85% of monocytes (versus ~40–50% with saline injection) were CD36^−^ monocytes (Figure 4B–D). Thus, P407 injection in Ldlr^−/−^ mice induced early and persistent profound reductions in the proportion of circulating CD36^+^, but not CD36^–^, monocytes. Of note, this change appeared to be specific for P407 injection because similar elevations in plasma TG in Ldlr^−/−^ mice induced by apoCIIItg compared to P407 injection were associated with significantly higher proportions of CD11c^+^ (CD36^+^) monocytes (Supplementary Figure S5).

### 3.5. P407 Did Not Increase Monocyte Apoptosis in Ldlr^−/−^ Mice

**Figure 5:**
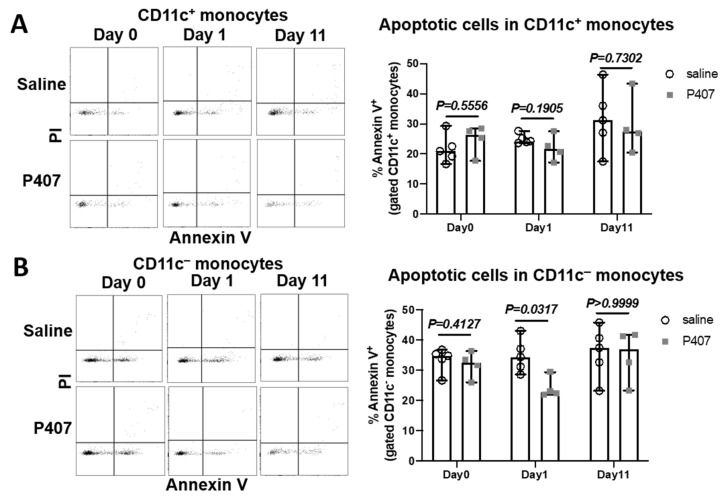
Monocyte apoptosis in Ldlr^−/−^ mice with P407 injection. FACS examples showing annexin V^−^PI^−^ (viable), annexin V^+^PI^−^ (early apoptotic), and annexin V^+^PI^+^ (late apoptotic) cells in (**A**) CD11c^+^ or (**B**) CD11c^−^ monocytes and quantitation of total apoptotic cells in (**A**) CD11c^+^ or (**B**) CD11c^−^ monocytes in Ldlr^−/−^ mice with P407 or saline injection. *n* = 4–5 mice/group. Data are shown as median with 95% confidence interval and were analyzed by the Mann–Whitney test.

A previous study showed that P407 injection in WT mice did not increase monocyte apoptosis; Ref. [21] therefore, the severe reduction in CD36^+^ (Ly-6c^low^) monocytes in WT mice with P407 injection may not arise from monocyte apoptosis. However, the authors examined monocyte apoptosis at time points when the numbers of Ly-6c^low^ monocytes were already low. To examine whether cell apoptosis was the main cause for reduced Ly-6c^low^ (CD36^+^) monocytes in Ldlr^−/−^ mice with P407 injection, we examined monocyte apoptosis early, on day 1, when this monocyte subset was not profoundly reduced, as well as on day 11, when this monocyte subset was greatly reduced. Based on the availabilities of the fluorophores that were conjugated to specific antibodies, we examined apoptosis in CD11c^+^ and CD11c^−^ monocytes, which mostly correspond to CD36^+^ and CD36^−^ monocytes, respectively, in Ldlr^−/−^ mice (Figure 3D). Indeed, injection of P407 versus the saline control did not increase apoptosis (annexin V^+^), including early (annexin V^+^PI^–^) and late (annexin V^+^PI^+^) apoptosis, of either CD11c^+^ or CD11c^−^ monocytes on day 1 and day 11 (Figure 5 and Supplementary Figure S6), consistent with the previous conclusion that the reduction in Ly-6c^low^ (CD11c^+^ and CD36^+^) monocytes in mice with P407 injection was not driven by increased cell apoptosis.

### 3.6. Infiltration of CD36^+^ Monocytes into Atherosclerotic Lesions in Ldlr^−/−^ Mice with P407 Injection Was Reduced Early and Persistently

**Figure 6:**
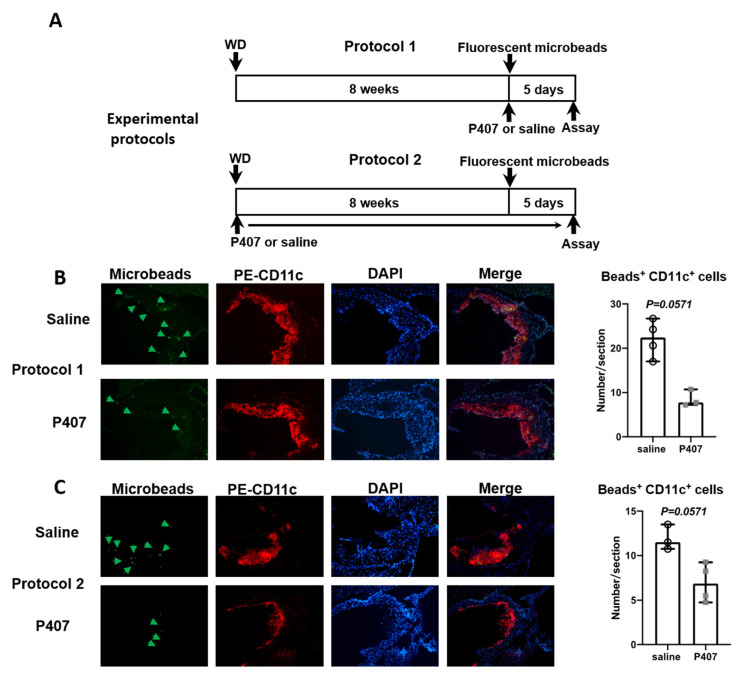
CD11c^+^ (CD36^+^) monocyte infiltration into atherosclerotic plaques. (**A**) Experimental protocols for WD feeding, P407 or saline injection, and injection of fluorescent microbeads (to label CD11c^+^ monocytes) in Ldlr^−/−^ mice. Representative images of aortic root showing infiltration of microbead (green)-labeled CD11c^+^ monocytes into atherosclerotic plaques and staining for CD11c (red), DAPI (blue), and quantitation of labeled CD11c^+^ monocyte infiltration into plaques in protocols (**B**) 1 and (**C**) 2. *n* = 3–4 mice/group. Data are shown as median with 95% confidence interval and were analyzed by the Mann–Whitney test.

The dramatic reduction in Ly-6c^low^ monocytes in the circulation of WT mice injected with P407 was attributed to increased extravasation of this monocyte subset into tissues including the liver, heart, and kidney [21]. Therefore, we examined whether P407 injection into Ldlr^−/−^ mice also altered Ly-6c^low^ (CD11c^+^ and CD36^+^) monocyte trafficking into atherosclerotic plaques. To this end, we followed the trafficking of this monocyte subset into atherosclerotic plaques in mice intravenously injected with fluorescent microbeads that specifically labeled Ly-6c^low^ (CD11c^+^ and CD36^+^) monocytes [12,28]. Due to the profound reduction in CD11c^+^ (CD36^+^) monocytes and also reduced atherosclerosis in Ldlr^−/−^ mice injected with P407, we used two experimental protocols (Figure 6A) to track CD11c^+^ (CD36^+^) monocyte trafficking into plaques.

In protocol 1 (Figure 6A), Ldlr^−/−^ mice were first fed the WD for ~8 weeks to induce atherosclerosis and then injected with P407 or the saline control along with a bolus of fluorescent microbeads. The numbers of monocytes, including CD11c^+^ (CD36^+^) monocytes, and the atherosclerotic lesion size were similar in the P407 and control groups at the time of microbead injection. At 24 h after microbead injection (and the first injection of P407 or the saline control), the number of circulating monocytes tended to be lower in the P407 group than the control group (Supplementary Figure S7A), consistent with the data in Figure 4. As expected, and consistent with previous reports, [12,28] injection of fluorescent microbeads specifically labeled CD11c^+^ monocytes in both the P407 and control groups (Supplementary Figure S7A). The proportion of CD11c^+^ monocytes labeled was moderately lower in the P407 group than the control group (Supplementary Figure S7A). Along with this change, the migration of CD11c^+^ monocytes into atherosclerotic plaques was reduced in the P407 group compared with the control group (Figure 6B).

In protocol 2 (Figure 6A), Ldlr^−/−^ mice were fed the WD and concurrently received injections of P407 or saline every other day for 8 weeks, when CD11c^+^ monocytes and atherosclerotic lesions were expected to be reduced in the P407 group compared with the control group (Figure 2 and Figure 4C), and a bolus of fluorescent microbeads was injected in both groups. As expected, at 24 h after microbead injection (8 weeks of P407 injection), the numbers of monocytes, mainly CD11c^+^ (CD36^+^) monocytes, were strikingly lower in the P407 group than the control group (Supplementary Figure S7B). For this reason, whereas microbeads consistently labeled CD11c^+^ monocytes in the control group, few monocytes were labeled in the P407 group (Supplementary Figure S7B). Analysis of atherosclerotic plaques showed that fewer CD11c^+^ monocytes infiltrated into plaques in the P407 group than in the control group (Figure 6C).

Thus, compared with controls, Ldlr^−/−^ mice with P407 injection had early and persistent reductions in CD11c^+^ (CD36^+^) monocyte infiltration into atherosclerotic plaques.

## 4. Discussion

Although HTG increases ASCVD risk, the underlying mechanisms are incompletely understood. Based on the role of monocytes in atherogenesis, this study sought to examine the effect of HTG induced by P407, an LPL inhibitor, on monocytes, and the potential role of monocytes in atherogenesis in Ldlr^−/−^ mice. Compared with the saline control, P407 induced rapid and persistent elevations in plasma TG and modest elevations in cholesterol levels in Ldlr^−/−^ mice. Unexpectedly, Ldlr^−/−^ mice had less atherosclerosis with P407 injection compared to saline alone. Circulating monocytes, mainly CD36^+^ (CD11c^+^) monocytes, had early increases in lipid accumulation, but subsequently, the proportion of circulating CD36^+^ monocytes was dramatically and persistently decreased, with reduced infiltration into atherosclerotic plaques in Ldlr^−/−^ mice injected with P407 compared to the saline control.

P407 directly inhibits the enzymatic activity of LPL; therefore, injection of P407 into mice produces severe HTG [22]. Unlike familial chylomicronemia syndrome, which is caused by genetic deficiency of LPL and characterized by severe HTG but very low LDL-C, mice with P407 injection have elevated LDL-C, [31] suggesting that P407 may have actions additional to LPL inhibition. Elevated TG and cholesterol in VLDL and LDL in Ldlr^−/−^ mice receiving P407 may also be due to LDLR deficiency, which impairs LDL clearance, accelerating elevations in plasma LDL-C and LDL-TG with P407 injection. P407 also inhibited the activity of endothelial lipase [32,33]. The reduction in HDL-C levels with P407 injection may be because of physical disruption of HDL by P407 or may indicate that inhibition of endothelial lipase may not play a major role in this mouse model with P407 injection. With these changes in plasma lipids, similar to mixed dyslipidemia in humans, we selected P407-treated Ldlr^−/−^ mice as a model for studying the effects of HTG on atherosclerosis in the context of hypercholesterolemia.

A previous study showed that WT mice injected with P407 for a long term (4–5 months) developed minor atherosclerotic lesions [22]. Our current study focused on early atherogenesis (WD for 7 weeks), in which monocyte recruitment may play a more important role [34,35]. The reduction in atherogenesis in Ldlr^−/−^ mice with P407-induced HTG as compared to Ldlr^−/−^ control mice was unexpected. Macrophage LPL has been implicated in atherogensis [36]. Therefore, inhibition of macrophage LPL by P407 may have contributed to reduced atherosclerosis with P407 injection. However, this effect is unlikely to be the major contributor in our study, given that HTG induced in Ldlr^−/−^ mice by transgenic expression of human apoC3, which also inhibits LPL activity, increased atherosclerosis, and that LPL-deficient mice developed atherosclerosis [37,38]. Of note, the reduction in atherogenesis in Ldlr^−/−^ mice with P407 injection was associated with an early and persistent decrease in the proportion of circulating monocytes, particularly CD36^+^ (CD11c^+^ and Ly-6c^low^) monocytes. Earlier studies showed contributions of Ly-6c^high^ monocytes to atherosclerosis [9,10,39]. More recent studies showed that Ly-6c^low^ (CD36^+^ and CD11c^+^) monocytes also played a pivotal role in atherogenesis, [8,12,40,41] and that infiltration of this monocyte subset into atherosclerotic plaques is positively associated with plaque size in mice [11,12,28,42,43,44]. Therefore, the profound decrease in circulating CD36^+^ monocytes, with reduced infiltration into atherosclerotic plaques, may contribute to the reduction in atherosclerosis in Ldlr^−/−^ mice receiving P407.

Prior to the severe reduction, circulating CD36^+^ monocytes in Ldlr^−/−^ mice with P407-induced HTG had increased lipid accumulation and elevated levels of CD11c, an adhesion molecule that mediates monocyte adhesion to the endothelium [8,12,45]. These changes simulate the phenotypes of monocytes in humans with HTG [15,16,17,18,19,20] and consistently support that HTG increases lipid accumulation in monocytes and alters monocyte phenotypes. The increase in monocyte lipid accumulation with P407 injection also suggests that monocytes can acquire circulating lipids independent of LPL, consistent with a previous report [46]. Following early lipid accumulation, the proportion of CD36^+^ monocytes was reduced to an extremely low level in the circulation of mice with P407 injection. Our current study and a previous study [21] showing that P407 injection did not increase monocyte apoptosis in Ldlr^−/−^ mice or WT mice indicate that the reduction of circulating CD36^+^ monocytes is not solely due to cell apoptosis. This observation also implies that the reduction of atherosclerosis in Ldlr^−/−^ mice with P407 injection is unlikely caused by lesion-cell apoptosis.

Strikingly, Saja et al. [21] showed that in WT mice with P407-induced HTG, Ly-6c^low^ (CD36^+^), but not Ly-6c^high^, monocytes had an increased crawling and retention behavior at the endothelial interface and extravasated into tissues including the liver, kidney, heart, and spleen. Extravasation of Ly-6c^low^ monocytes, which is associated with tissue accumulation of lipid-laden foam macrophages, may underlie the reduction in Ly-6c^low^ (CD36^+^) monocytes in the circulation of WT mice with P407-induced HTG [21]. Based on this observation, we had expected that P407 injection would increase CD36^+^ monocyte trafficking into atherosclerotic plaques in Ldlr^−/−^ mice. However, contrary to our expectation, reduced infiltration of CD36^+^ (CD11c^+^) monocytes into atherosclerotic plaques was observed, beginning early after P407 injection, and persisting in Ldlr^−/−^ mice during P407 injection. The severe (in protocol 2) or modest (in protocol 1) reduction in the proportion of CD36^+^ monocytes may explain the decreased infiltration of this monocyte subset into atherosclerotic plaques in mice with P407 injection. In addition, given that blood flow is much faster in aortas than in other tissues such as the liver, spleen, and kidney, increased extravasation of CD36^+^ (CD11c^+^) monocytes into these other tissues may (competitively) prevent this monocyte subset from infiltrating into aortic walls. In support of this hypothesis, we observed that Ldlr^−/−^ mice with P407 injection had increased CD11c levels, indicating increased accumulation of CD11c^+^ cells in the liver (Supplemental Figure S8), possibly because of accelerated CD11c^+^ monocyte trafficking into the liver. Therefore, increased CD11c^+^ monocyte competitive trafficking into the liver and other tissues, leading to profound reductions in the proportion of circulating CD11c^+^ monocytes and decreased trafficking of CD11c^+^ monocytes into arterial walls, may contribute to the reduction in atherosclerosis in Ldlr^−/−^ mice with P407-induced HTG.

Our study has several limitations or weaknesses. First, because of the confounding effects of estrus on lipid metabolism, [23,24,25] only male mice were used, and we cannot apply the same observations to female mice. Second, although our and previous studies [21,22] suggest that P407 does not adversely affect mice or increase cell death, we cannot fully exclude a potentially direct effect of P407 on our outcomes. Third, P407 injection compared with the saline control caused a slight reduction in WD-induced weight gain, with smaller fat pads, in Ldlr^−/−^ mice. This can be explained by P407-mediated inhibition of LPL, resulting in decreased lipid transfer from the circulation into adipose tissue. Nevertheless, we cannot fully exclude a potential role of less weight gain in our observations. In addition, compared to apoCIIItgLdlr^−/−^ mice, another commonly used mouse model of HTG, Ldlr^−/−^ mice with P407-induced HTG showed a much more profound reduction in CD11c^+^ monocytes, suggesting that the profound reduction in CD11c^+^ monocytes is specific to P407 injection and may not be caused by HTG per se; thus, results collected from Ldlr^−/−^ mice with P407-induced HTG may not fully apply to other mouse models of HTG. Despite these limitations, our study revealed an unexpected finding of reduced atherogenesis in Ldlr^−/−^ mice with P407 injection and supported a potential role for altered CD11c^+^ (CD36^+^) monocytes in this process.

In summary, our study demonstrated that P407 injection in Ldlr^−/−^ mice induced severe HTG and mild elevations in plasma cholesterol levels, including LDL-C, which were associated with an early increase in lipid accumulation and CD11c levels in circulating monocytes, particularly CD36^+^ (CD11c^+^ and Ly-6^low^) monocytes. However, Ldlr^−/−^ mice with P407 injection showed reduced atherosclerosis, possibly because of severe reductions in the proportion of circulating CD36^+^ monocytes, leading to reduced infiltration of CD36^+^ monocytes into atherosclerotic plaques.

## Supplementary Materials

The following supporting information can be downloaded at: https://www.mdpi.com/article/10.3390/cells11111795/s1. Figure S1: Body weight and weight of epididymal adipose tissue and liver; Figure S2: Lesion size in the brachiocephalic artery (BCA) of Ldlr^−/−^ mice with P407 or saline administration; Figure S3: Representative FACS examples of circulating monocytes showing higher side scatter (SSC), indicating more lipid accumulation, of circulating monocytes in Ldlr^−/−^ mice with P407 injection (for 1 and 3 days) than with saline injection; Figure S4: Effects of mouse plasma on THP1 cell lipid accumulation; Figure S5: Plasma TG and proportions of CD11c+ monocytes. Ldlr^−/−^ mice (Con) or Ldlr^−/−^ mice with human apoCIII transgenic expression (apoCIIItg) were fed WD for 7 weeks, or Ldlr^−/−^ mice fed WD were injected with P407 (P407) every other day for 7 weeks. Plasma total TG levels were measured by enzymatic methods. CD11c+ (CD36+) monocytes were examined by FACS analysis and presented as percentages in total monocytes; Figure S6: Apoptosis of circulating monocytes in Ldlr^−/−^ mice with P407 injection; Figure S7: Labeling of CD11c+ monocytes by injecting fluorescent microbeads in Ldlr^−/−^ mice with P407 or saline administration; Figure S8: CD11c mRNA in the liver of Ldlr^−/−^ mice with P407 or saline administration; Major Resources Table S1.

## Nonstandard Abbreviations and Acronyms

ASCVD	atherosclerotic cardiovascular disease
BCA	brachiocephalic artery
CM	chylomicrons
HTG	hypertriglyceridemia
LDL	low-density lipoprotein
LDL-C	low-density lipoprotein cholesterol
LDL-TG	low-density lipoprotein–triglyceride
LPL	lipoprotein lipase
P407	poloxamer 407
PI	propidium iodide
SEC	size-exclusion chromatography
SSC	side scatter
TG	triglyceride
VLDL	very-low-density lipoprotein
WD	Western high-fat high-cholesterol diet
WT	wild-type
