# Supplementary material for: Poloxamer 407 Induces Hypertriglyceridemia but Decreases Atherosclerosis in Ldlr−/− Mice
Source: Cells. 2022 May 30;11(11):1795. doi: 10.3390/cells11111795 (PMC9179832; doi:10.3390/cells11111795)
Supplement: Supplementary file 1 [file cells-11-01795-s001.zip › Supplementary Figures S1-S8 .pdf]

Poloxamer 407 induces hypertriglyceridemia but decreases atherosclerosis in Ldlr$^{-/-}$ mice

SUPPLEMENTAL MATERIALS

Supplementary Figures S1-S8

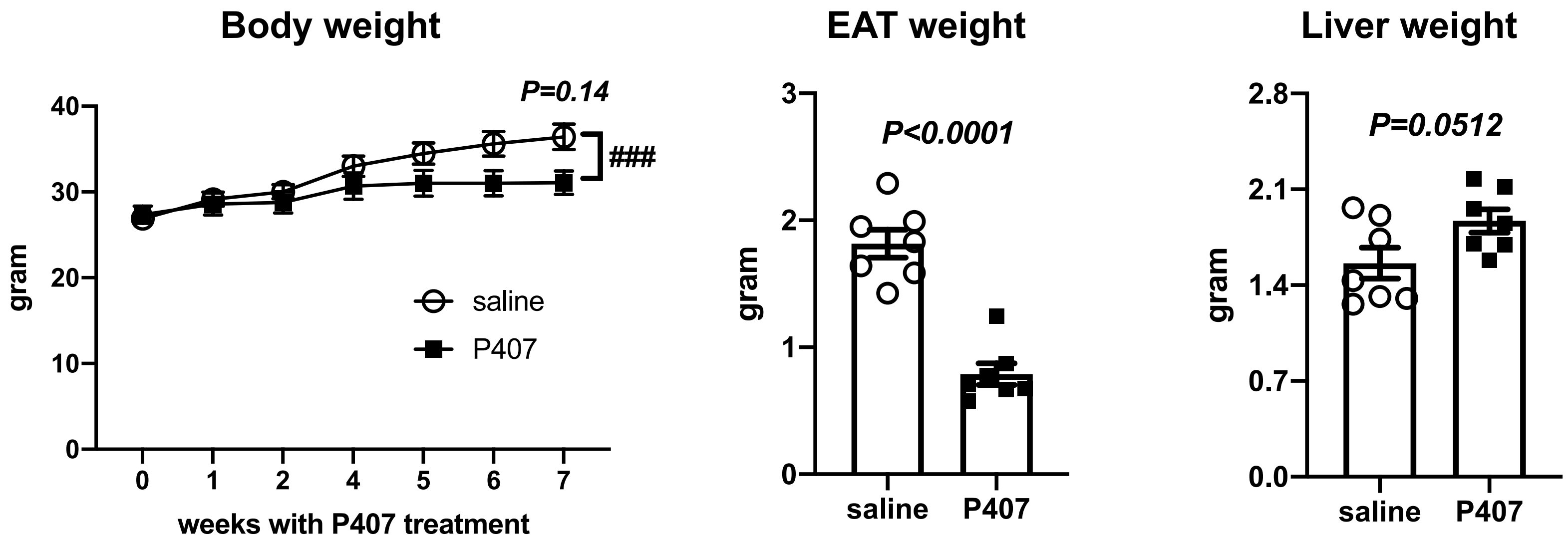

Supplemental Figure S1. Body weight and weight of epididymal adipose tissue and liver. Ldlr$^{-/-}$ mice were fed western high-fat high-cholesterol diet and injected with P407 or saline for 7 weeks. Changes in body weight were monitored weekly. Epididymal adipose tissue (EAT) and liver were weighed at 7 weeks of P407 or saline injection. n=7 mice/group. Data are shown as mean±SEM and were analyzed by 2-way ANOVA with repeated measures and assumption of inequivalence of variance followed by Šídák's multiple pairwise comparisons test (for body weight) or by unpaired Student's t-test (for EAT and liver weight). ###P<0.001 for group x time interaction by 2-way ANOVA.

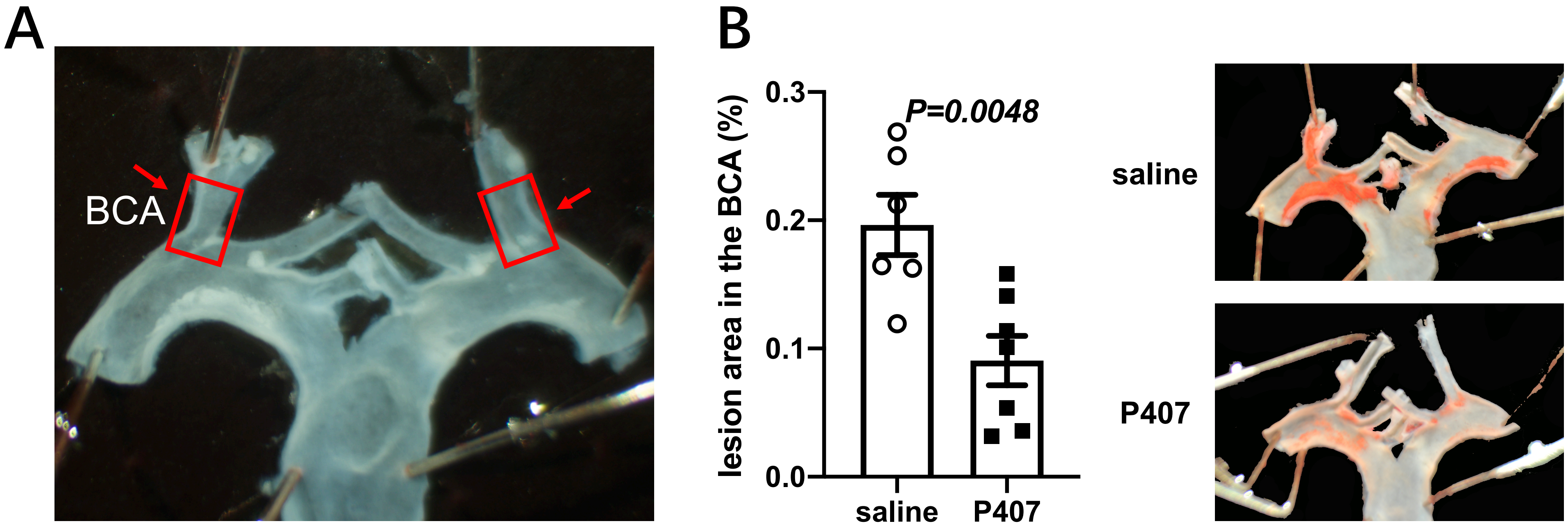

Supplemental Figure S2. Lesion size in the brachiocephalic artery (BCA) of Ldlr$^{-/-}$ mice with P407 or saline administration. (A) Overview of aortic arch. Red rectangles indicate region of interest for plaque analysis in the BCA. (B) Atherosclerotic lesion in
the BCA and representative images of aortic arch with oil red O staining in saline and P407 groups. Data are shown as mean±SEM and were analyzed by unpaired Student's t-test.

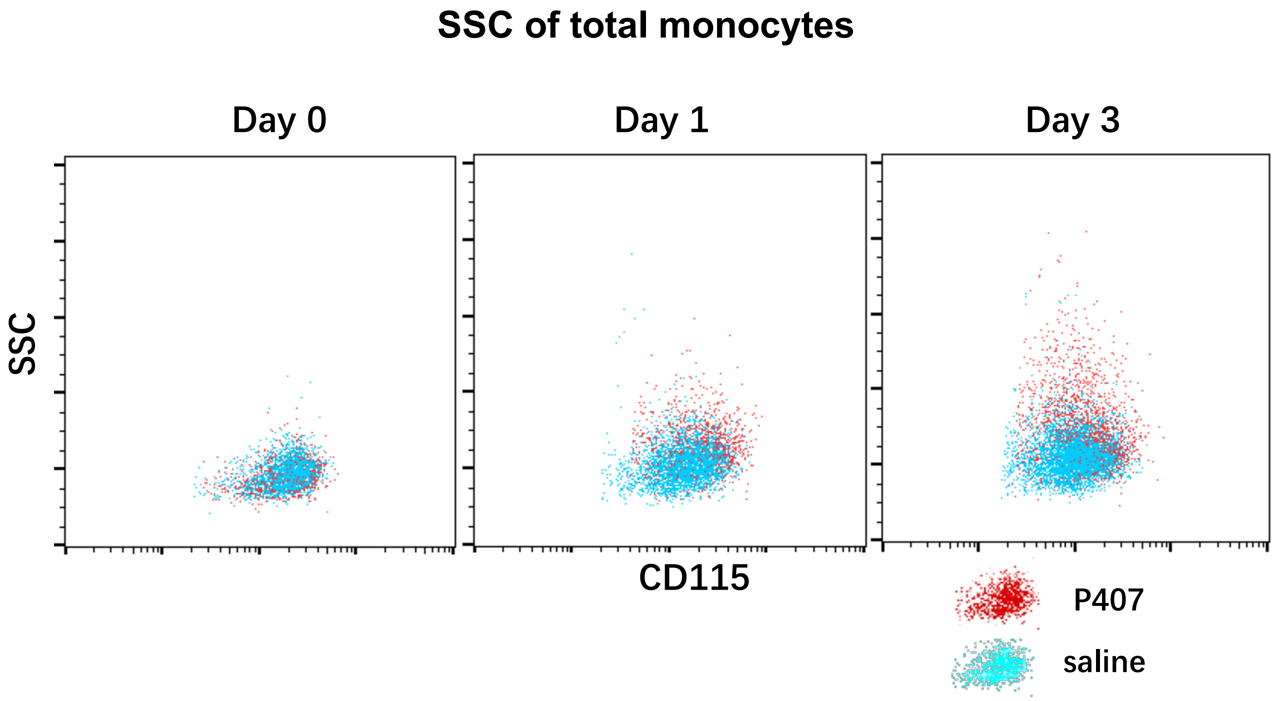

Supplemental Figure S3. Representative FACS examples of circulating monocytes showing higher side scatter (SSC), indicating more lipid accumulation, of circulating monocytes in Ldlr$^{-/-}$ mice with P407 injection (for 1 and 3 days) than with saline injection.

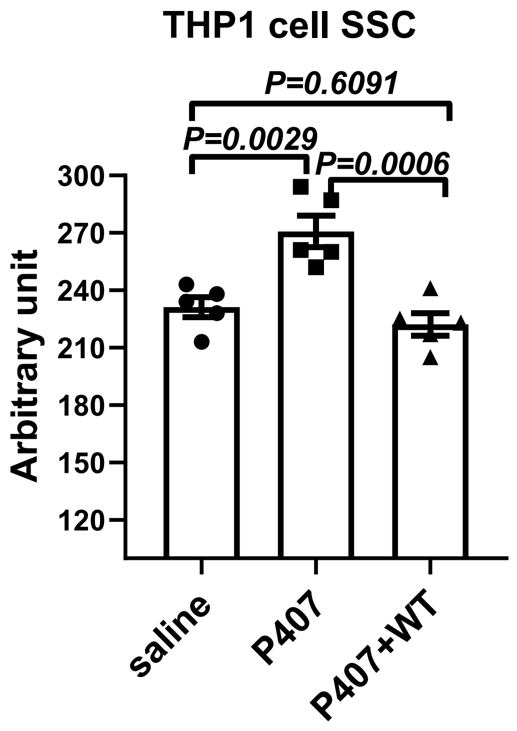

Supplemental Figure S4. Effects of mouse plasma on THP1 cell lipid accumulation. THP1 cells were cultured in RPMI-1640 medium supplemented with 30% (v/v) plasma from Ldlr$^{-/-}$ mice injected with saline (saline) or P407 (P407) or plasma from P407-injected Ldlr$^{-/-}$ mice plus plasma from WT mice (P407+WT, to match the TG level in saline group). After 48 hours, THP1 cells were examined by FACS analysis for lipid accumulation as indicated by SSC values. Data are shown as mean±SEM and were analyzed by 1-way ANOVA followed by multiple pairwise comparisons test.

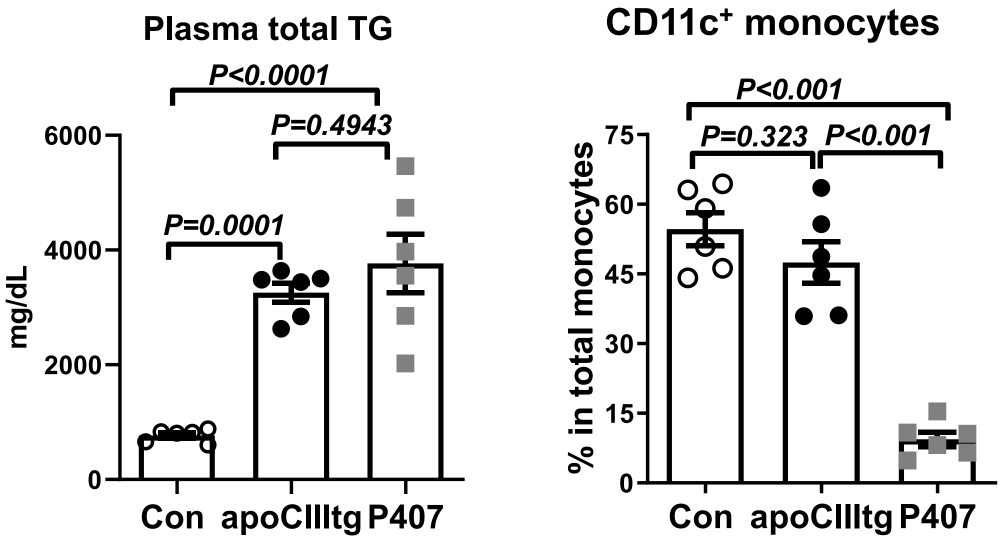

Supplemental Figure S5. Plasma TG and proportions of CD11c$^+$ monocytes. Ldlr$^{-/-}$ mice (Con) or Ldlr$^{-/-}$ mice with human apoCIII transgenic expression (apoCIIItg) were fed WD for 7 weeks, or Ldlr$^{-/-}$ mice fed WD were injected with P407 (P407) every other day for 7 weeks. Plasma total TG levels were measured by enzymatic methods. CD11c$^+$ (CD36$^+$) monocytes were examined by FACS analysis and presented as percentages in total monocytes. Data are shown as mean±SEM and were analyzed by 1-way ANOVA followed by multiple pairwise comparisons test.

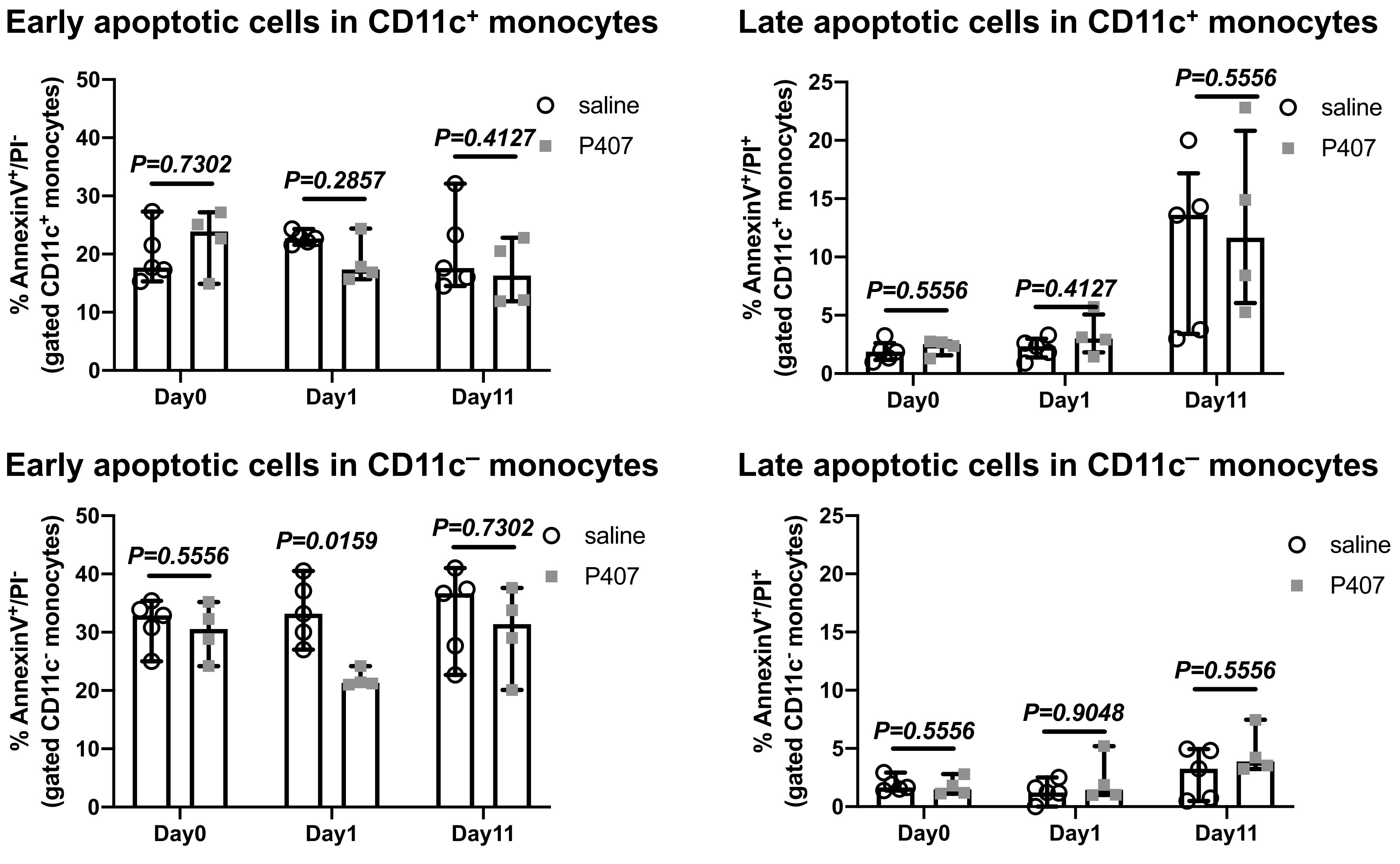

Supplemental Figure S6. Apoptosis of circulating monocytes in Ldlr$^{-/-}$ mice with P407 injection. Blood samples from Ldlr$^{-/-}$ mice with P407 or saline injection were stained with FITC–annexin V, PE–propidium iodide (PI), PE-Cy7–CD115, and APC–CD11c, and then examined by FACS analysis. Percentages of annexin V^{+}PI^{-} (early) and annexin V^{+}PI^{+} (late) apoptotic cells in CD11c^{+} and CD11c^{-} monocytes are presented. n=4–5 mice/group. Data are shown as median with 95% confidence interval and were analyzed by Mann–Whitney test.

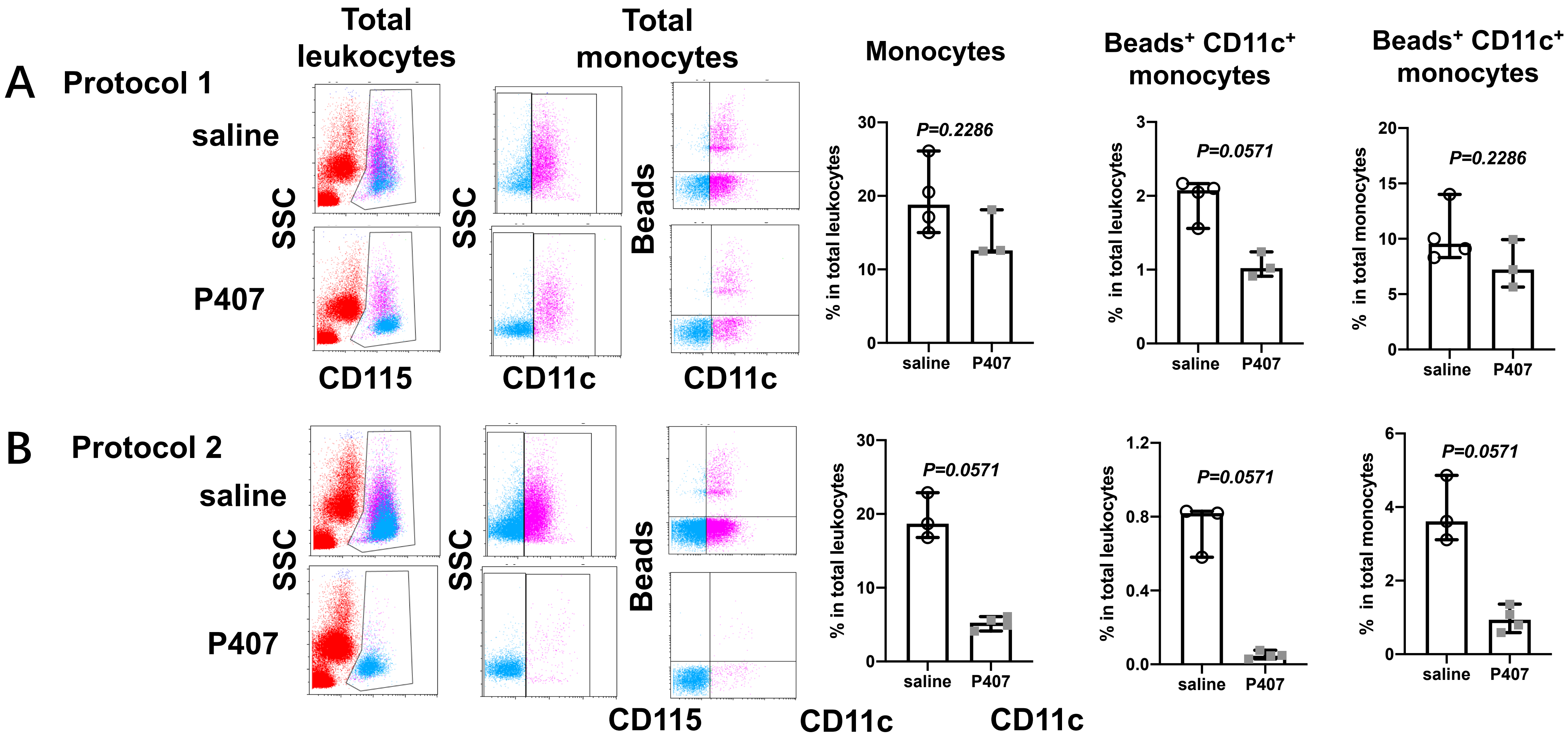

Supplemental Figure S7. Labeling of CD11c$^+$ monocytes by injecting fluorescent microbeads in Ldlr$^{-/-}$ mice with P407 or saline administration.(A) In protocol 1, Ldlr$^{-/-}$ mice were fed WD for around 8 weeks to induce atherosclerosis and then injected with P407 or saline and simultaneously received injection of fluorescent microbeads. At 24 hours after the injection, FACS analysis showed labeling of CD11c$^+$ monocytes by microbeads and modest reductions in monocytes and bead-labeled CD11c$^+$ monocytes in the P407 group. (B) In protocol 2, Ldlr$^{-/-}$ mice were fed WD and simultaneously received P407 or saline injection every other day for 8 weeks and then injected with fluorescent microbeads. At 24 hours after microbead injection, FACS analysis showed labeling of CD11c$^+$ monocytes by microbeads (mainly in the saline group) and profound reductions in CD11c$^+$ monocytes and monocyte labeling in the P407 group. Data are shown as median with 95% confidence interval and were analyzed by Mann–Whitney test.

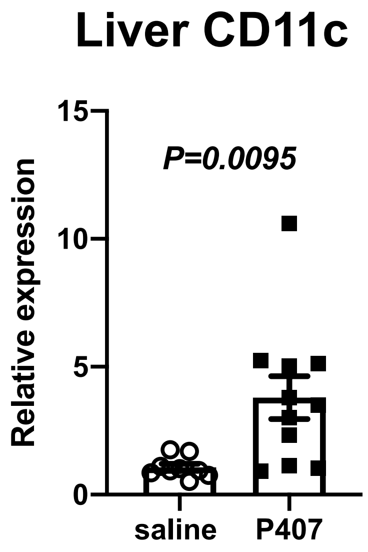

Supplemental Figure S8. CD11c mRNA in the liver of Ldlr$^{-/-}$ mice with P407 or saline administration. mRNA levels of CD11c were examined by quantitative RT-PCR in the liver of Ldlr$^{-/-}$ mice with P407 or saline administration for 7 weeks. n=9–12 samples/group. Data are shown as mean±SEM and were analyzed by unpaired Student's t-test.
